# Dental caries and mean values of DMFT among children with cerebral palsy: a systematic review and meta-analysis

**DOI:** 10.1186/s12903-024-03985-5

**Published:** 2024-02-15

**Authors:** Melkamu Aderajew Zemene, Anteneh Mengist Dessie, Denekew Tenaw Anley, Mengesha Assefa Ahunie, Natnael Atnafu Gebeyehu, Getachew Asmare Adella, Gizachew Ambaw Kassie, Misganaw Asmamaw Mengstie, Mohammed Abdu Seid, Endeshaw Chekol Abebe, Molalegn Mesele Gesese, Natnael Amare Tesfa, Yenealem Solomon Kebede, Natnael Moges, Berihun Bantie, Sefineh Fenta Feleke, Tadesse Asmamaw Dejenie, Wubet Alebachew Bayih, Ermias Sisay Chanie

**Affiliations:** 1 Department of Public Health, College of Health Sciences, Debre Tabor University, Debre Tabor, Ethiopia; 2 Department of Midwifery, College of Medicine and Health Science, Wolaita Sodo University, Wolaita Sodo, Ethiopia; 3 Department of Reproductive Health and Nutrition, School of Public Health, Woliata Sodo University, Wolaita Sodo, Ethiopia; 4 Department of Epidemiology and Biostatistics, School of Public Health, Woliata Sodo University, Wolaita Sodo, Ethiopia; 5 Department of Biochemistry, College of Health Sciences, Debre Tabor University, Debre Tabor, Ethiopia; 6 Unit of Physiology, Department of Biomedical Science, College of Health Science, Debre Tabor University, Debre Tabor, Ethiopia; 7 School of Medicine, College of Health Science, Woldia University, Woldia, Ethiopia; 8 Department of Medical Laboratory Science, College of Health Sciences, Debre Tabor University, Debre Tabor, Ethiopia; 9 Department of Pediatrics and Child Health Nursing, College of Health sciences, Debre Tabor University, Debre Tabor, Ethiopia; 10 Department of Comprehensive Nursing, College of Health Sciences, Debre Tabor University, Debre Tabor, Ethiopia; 11 Department of Public Health, College of Health Sciences, Woldia University, Woldia, Ethiopia; 12 Department of Medical Biochemistry, College of Medicine and Health Sciences, University of Gondar, Gondar, Ethiopia; 13 Department of Maternal and neonatal Health Nursing, College of Health Sciences, Department of Epidemiology and preventive Medicine, School of Public Health and Preventive Medicine, Faculty of Medicine, Nursing and Health Sciences, Debre Tabor University, Monash University, Melbourne, VIC Australia

**Keywords:** Dental caries, Cerebral palsy, Oral health, Child, Africa, Asia

## Abstract

**Introduction:**

One of the most prevalent causes of physical disability in children is cerebral palsy (CP), which is a series of complicated neurological disorders. Children with cerebral palsy suffer from multiple problems and potential disabilities, including dental caries. Hence, this study aimed to determine the pooled prevalence of dental caries and mean DMFT (Decayed, Missed, and Filled Permanent Teeth) among children with cerebral palsy in Africa and Asia.

**Methods:**

A comprehensive search of the literature was made to locate relevant studies in PubMed/Medline, HINARI, Web of Science, Science Direct, the Cochrane Library, the Worldwide Science Database, and Google Scholar. The data were extracted in Microsoft Excel and transferred to Stata version 17 software for further analysis. A random-effect model was employed to estimate the pooled prevalence of dental caries and the pooled mean value of DMFT among children with cerebral palsy in Africa and Asia. Heterogeneity between studies was checked using the Cochrane Q test and I^2^ test statistics. Sub-group analysis by continent was done, and sensitivity analysis was checked. A small study effect was checked using Egger’s statistical test at the 5% level of significance.

**Results:**

In this study, 25 original studies conducted in 17 countries in Africa and Asia that fulfilled the eligibility criteria were included in the review. The overall pooled prevalence of dental caries in Africa and Asia among children with cerebral palsy was 55.6% (95% CI: 42.4, 68.8). The pooled prevalence of dental caries among children with cerebral palsy in Africa was 42.43% (95% CI: 30.39, 54.58), and it was slightly higher in Asia with 64% (95% CI: 48.32, 79.72). In the random effect model analysis, the pooled mean DMFT of dental caries in children with cerebral palsy was 2.25 (95% CI: 1.86, 2.64). The pooled mean DMFT in Africa was 1.47 (95% CI: 0.86, 2.09), and in Asia it was 3.01 (95% CI: 2.43, 3.60).

**Conclusion:**

In this study, we found that children with cerebral palsy experienced an alarming rate of dental caries. In these settings, dental caries affected roughly more than half of the children with cerebral palsy. Hence, oral health promotion initiatives should target children with CP, and this group of children must receive early preventive dental care.

**Supplementary Information:**

The online version contains supplementary material available at 10.1186/s12903-024-03985-5.

## Introduction

Cerebral palsy (CP) is defined as a group of complex neurological disorders caused by non-progressive injury to the developing brain, which leads to abnormalities in movement and posture [1]. The level of intellectual disability of the child with CP is different [2]. The majority are intellectually normal or have mild to moderate disabilities. About 25% of children with cerebral palsy have a severe intellectual disability [3]. This motor dysfunction typically results in changes to cognition, sensation, behavior, and communication [4]. Globally, one billion people, or 15% of the world’s population experience disabilities [5]. Of these, 29.2% attributed to common mental disorders [6]. The overall median prevalence of cerebral palsy was estimated at 2.4 per 1000 live births [7].

The global prevalence of dental caries from the overall children in permanent dentition was estimated to be 35% [8]. However, the problem is much higher in children with cerebral palsy. Children with cerebral palsy are partially, if not completely dependent on the caregiver to conduct regular duties like feeding, moving around, and maintaining oral and general hygiene [9]. The food they eat, their eating patterns, medications, physical restrictions, lack of cleaning habits, and attitudes of parents and healthcare providers, are the factors that contribute to poor dental health in this group population [10].

Oral disease is one of the major public health problems for individuals with disabilities, particularly in children with cerebral palsy. Children with cerebral palsy are more likely to experience severe morbidity from dental issues, which can impair their overall health and further worsen their quality of life [11]. Poor oral health in children with cerebral palsy is indicated by high rates of dental caries, missing teeth, periodontal disease, prolonged primary teeth retention, misaligned or extra teeth, and malocclusion [12]. Poor oral hygiene can have several negative effects on a person’s overall health, including tooth decay, gum disease, bad breath, tooth loss, and oral infections. Besides, it has also been linked to several health problems, including heart disease, stroke, diabetes, and respiratory infections. Moreover, poor dental health hurts aesthetics, speech, face structure, digestion, chewing, and enjoyment of food [13].

Children with cerebral palsy may have a higher risk of developing bruxism or clenching, which can lead to tooth damage and decay. Difficulty in swallowing and controlling saliva also increases the risk of tooth decay. Dental caries among children with cerebral palsy is worsened by not only the high incidence rate but also the significant burden of untreated dental caries [14].

Access to healthcare has been also a problem for children with cerebral palsy. Despite the high prevalence of dental caries among children with cerebral palsy in Africa and Asia, healthcare service is the most common unmet need for this group of individuals [15]. Their oral health needs may have been also compromised due to parental neglect or the disregard shown by most dental and medical experts. Moreover, limited access to dental care, low socioeconomic status, limited awareness and education, and dietary factors contribute to the high burden of dental caries in low-and middle-income countries. The state of one’s oral health has significant psychological, biological, and social consequences to the affected person, their parents, or caregivers [16].

Therefore, determining the oral health condition of children with disabilities particularly children with cerebral palsy may help to plan preventative and therapeutic actions and lower treatment costs. Understanding the specific needs and barriers faced by these children can help in developing tailored oral health interventions and resources. Moreover, there is a lack of comprehensive data and evidence-based guidelines for oral health care for children with disabilities. As a result, more research can help fill this knowledge gap and provide valuable insights for oral health professionals and caregivers. Additionally, it is crucial for resource planning and creating community services tailored to the requirements of these disadvantaged people. Hence, this systematic review and meta-analysis aimed to determine the pooled prevalence and mean DMFT of dental caries among children with cerebral palsy in 17 countries from Africa and Asia.

## Methods and materials

### Study protocol registration

The protocol of this study was registered in the International Prospective Register of Systematic Reviews (PROSPERO) database and can be accessed with protocol number CRD42023430624.

### Design and searching strategy

A Comprehensive search of the literature was made in PubMed/Medline, HINARI, Web of Science, Science Direct, Cochrane Library, and, the worldwide Science database. In addition, manual search and other grey literature from Google Scholar, Google, and reference lists of all relevant studies were screened. The search strategy was built by keywords for condition, context, and population using Boolean terms. As a result, the MeSH terms “(((((((“Dental caries“[MeSH Terms])) OR (“Tooth decay” [MeSH Terms])) OR (Decay [MeSH Terms])) OR (“Dental health“[MeSH Terms])) OR (“Oral health“[MeSH Terms])) AND (Children [MeSH Terms])) AND (“Cerebral palsy“[MeSH Terms])) AND (Africa [MeSH Terms])) AND (Asia [MeSH Terms])” were used for PubMed. The search combinations were adapted for use in other databases. Two author groups, Group One (MAZ & ESC) and Group Two (AMD & DTA), searched the articles independently up to May 31/2023, using the above search combinations.

### Eligibility criteria

Primary studies that reported the prevalence of dental caries or the mean value of DMFT among children with cerebral palsy in countries from Africa or Asia were included. All the available and eligible studies were observational studies published in English. We excluded reviews of review, qualitative studies, and studies with incomplete information or did not report the outcome of interest.

### Selection and data extraction

A total of 1303 studies identified from the literature search were exported into Endnote X8 software. All duplicate studies were examined and removed. Titles and abstracts were independently screened for inclusion in the full-text appraisal by the two groups of review authors. We created a data extraction format in Microsoft Excel to extract all the essential data. The data extraction format included first author, year of publication, continent, country, study design, sample size, prevalence, and/or mean and Standard Deviation (SD) of DMFT. If there was a disagreement among the investigators, the study was reassessed using the predefined inclusion criteria until a consensus was reached by all of the members. The study selection process was shown graphically using a PRISMA flow chart [17].

### Quality assessment

The quality of each original study was assessed using the Newcastle Ottawa Scale (NOS) for assessing non-randomized studies [18]. The scale evaluates case-control studies with a maximum of nine stars using a three-part approach; the selection (4 points), comparability (2 points), and the exposure/outcome (3 points). Similarly, an adapted NOS scale was used to assess the risk of bias in cross-sectional studies with a maximum of ten stars [19]. High-quality studies are defined by NOS score of > 5 stars, while a NOS score of ≤ 5 stars indicates a low-quality study. Two groups of reviewers: Group One (MAZ, ESC) and Group Two (AMD, DTA) independently assessed the study quality. Differences between the two groups were resolved through discussion until reached on consensus or the decision was determined by the third group of review authors (YSK, NM).

### Outcome measurement

According to the WHO’s 2013 oral health assessment tool for children, dental caries were assessed using the decayed, missing, and filling index for caries measurement (DMFT) [20]. A tooth was considered decayed when there was apparent carious cavitation on any surface of the tooth. A tooth was classified as missing in the index if it was extracted due to caries. A tooth was classified as filled if it had a restoration for a carious lesion.

### Data analysis

The extracted data were exported from a Microsoft Excel spreadsheet to STATA version 17 (MP) for further management and analysis. The heterogeneity between the prevalence of the prior studies was examined using the Cochrane Q-test and I^2^ test statistics. The studies’ heterogeneity was classified as low, moderate, and high based on the I^2^ values of less than 50%, 50-75%, and above > 75%, respectively. Due to the presence of significant heterogeneity (I^2^ = 98.8%, *p* < 0.001), a random-effects model (Der Simonian and Laird’s) was used to estimate the pooled prevalence of dental caries among children with cerebral palsy in Africa and Asia. To minimize the variance of estimated points between primary studies, a sub-group analysis by the continent was done. A sensitivity analysis was also conducted to determine the influence of a single study on the pooled prevalence. Moreover, univariate meta-regression was also done by using the year of publication, sample size, and country using the random effect models. Any potential publication bias was evaluated using the visual funnel plot test and Egger’s test at a 5% significant level. The results were presented in text, table, graph, and Forest plot.

## Results

### Searching results

**Fig. 1:**
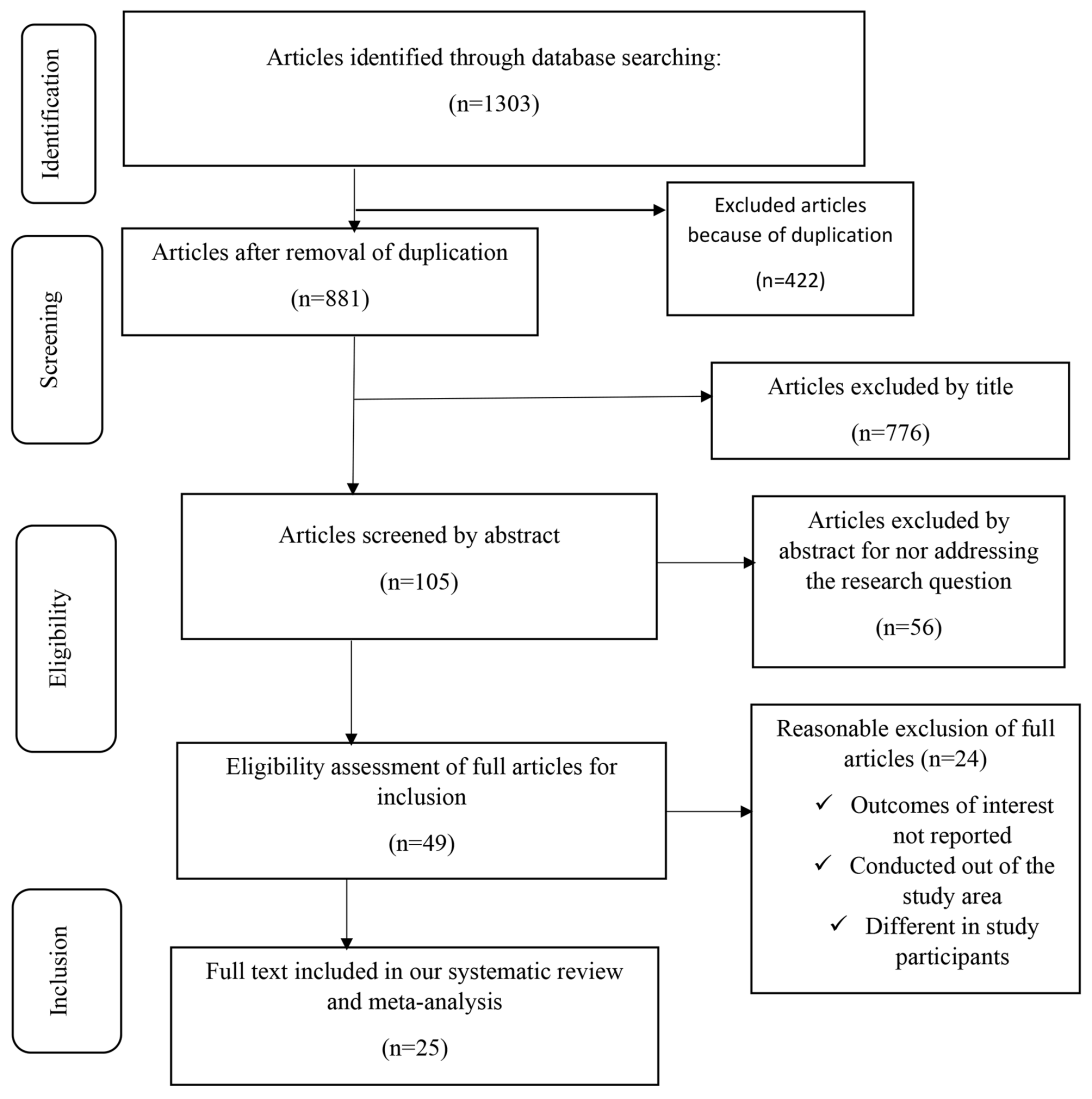
Flow chart diagram describing selection of studies for the systematic review and meta-analysis of dental caries among children with cerebral palsy in 17 countries from Africa and Asia

We identified 1303 studies from Medline, HINARI, Web of Science, Science Direct, Cochrane Library, worldwide Science database, and Google Scholar. Of these, 422 records were excluded due to duplication. Then, non-pertinent articles were excluded through reading the titles, abstracts, and full texts. Reasons for exclusion were mainly due to not addressing the research question, lack of report on the outcome of interest, studies being conducted out of the area for this review, and use of different study participants. The kappa value for the group of researchers was 0.91 and 0.96 at abstract and full-text screening respectively, indicating adequate agreement between the two reviewers. Finally, 25 studies with an overall quality score of greater than or equal to six were included in the systematic review and meta-analysis. Details of the study selection algorithm is presented in Fig. 1.

### Descriptions of included studies

Overall, we included 18 cross-sectional and 7 case-control studies in the review. Of these, ten studies were from African countries including Nigeria [21–24], Egypt [25, 26], Cameroon [27], South Africa [28], Uganda [29], and Sudan [30]. Other studies were from Asian countries including Bangladesh [31], Saudi Arabia [32–34], India [35–37], Thailand [38], China [39], United Arab Emirates [40], Sri Lanka [41], Turkey [42], Iran [43], Pakistan [44], and Jordan [45]. Studies from 2008 to 2022 were included. The pooled study population included 1754 children with cerebral palsy from 18 cross-sectional studies and 912 children (445 children with cerebral palsy and 467 controls) from 7 case-control studies. About 56% of studies had a sample size of > 70. The highest prevalence of dental caries among children with cerebral palsy was reported in Saudi Arabia (98.5%) [32], whereas the lowest was from Sri Lanka (8.6%) [41] (Table 1). All of the included studies were of a high-quality range with a NOS score of 6 to 8 stars. The risk of bias within studies based on the NOS criteria is presented in Table 2.

**Table 1:** The mean value of DMFT and prevalence of dental caries distribution among children with cerebral palsy in 17 countries from Africa and Asia

First author/year	Country	Continent	Study design	Sample size	Prevalence (%)	Mean value of DMFT (SD)
Folaranmi et al. (2014)	Nigeria	Africa	Cross-sectional	42	47.6	0.48 ± 2.74
Sedky et al. (2017)	Egypt	Africa	Cross-sectional	62	54.8	4.18 ± 5.6
Akhter et al. (2017)	Bangladesh	Asia	Cross-sectional	90	52.2	0.7 ± 1.79
Alhammad et al. (2010)	Saudi Arabia	Asia	Cross-sectional	140	98.6	20.9 ± 16
Nqcobo et al. (2012)	South Africa	Africa	Cross-section	163	56.4	0.68 ± 2.03
Sinha et al. (2015)	India	Asia	Case-control	50	Not found	4.11 ± 2.62
Quritum et al. (2022)	Egypt	Africa	Case-control	80	Not found	1.32 ± 1.73
Wyne et al. (2017)	Saudi Arabia	Asia	Cross-sectional	52	98.1	1.69 ± 2.72
Oredugba et al. (2008)	Nigeria	Africa	Case-control	54	33.3	0.4 ± 1.44
Pansrimangkorn et al. (2023)	Thailand	Asia	Cross-sectional	60	91.7	20.3 ± 21.6
Rose et al. (2022)	Cameroon	Africa	Cross-sectional	60	28.3	Not found
Ruchi et al. (2021)	India	Asia	Cross-sectional	100	57	Not found
Dalvand et al. (2021)	Iran	Asia	Cross-sectional	123	71.5	Not found
Nouri et al. (2015)	Saudi Arabia	Asia	Case-control	60	65	5.12 ± 7.38
Du et al. (2010)	China	Asia	Case-control	72	43.1	4.81 ± 11.24
Al Hashmi et al. (20,017)	UAE	Asia	Case-control	84	52.4	2.83 ± 2.86
Kachwinya et al. (2022)	Uganda	Africa	Cross-sectional	90	63.3	3.8 ± 4.5
Denloye et al. (2012)	Nigeria	Africa	Cross-sectional	61	11.48	0.07
Daraniyagala et al. (2019)	Sri Lanka	Asia	Cross-sectional	93	8.6	3.74 ± 5.01
Altun et al. (2010)	Turkey	Asia	Cross-sectional	136	84.5	1.62 ± 2.98
Nzomiwu et al. (2022)	Nigeria	Africa	Cross-sectional	81	40.7	0.4 ± 1.0
Hadeya et al. (2017)	Sudan	Africa	Cross-sectional	123	46.3	2.0 ± 2.9
Vandal et al. (2018)	India	Asia	Case-control	45	55.5	0.99 ± 0.08
Jawed et al. (2020)	Pakistan	Asia	Cross-sectional	196	58.2	2.05 ± 2.51
Aburahma et al. (2021)	Jordan	Asia	Cross-sectional	83	57.8	0.4 ± 0.6

**Table 2:** Detailed risk of bias according to the Newcastle Ottawa Scale (NOS)

Author	Selection	Comparability	Exposure/outcome	NOS score (out of 9 for case-control and 10 foe cross-sectional studies)
Folaranmi et al. (2014)	***	**	***	8
Sedky et al. (2017)	****	*	***	8
Akhter et al. (2017)	***	**	***	8
Alhammad et al. (2010)	****	**	*	7
Nqcobo et al. (2012)	***	**	**	7
Sinha et al. (2015)	***	**	*	6
Quritum et al. (2022)	***	**	*	6
Wyne et al. (2017)	***	**	**	7
Oredugba et al. (2008)	***	**	***	8
Pansrimangkorn et al. (2023)	***	**	**	7
Rose et al. (2022)	***	**	*	6
Ruchi et al. (2021)	****	*	*	6
Dalvand et al. (2021)	***	**	*	6
Nouri et al. (2015)	***	**	**	7
Du et al. (2010)	***	**	***	8
Al Hashmi et al. (20,017)	***	**	***	8
Kachwinya et al. (2022)	****	*	***	8
Denloye et al. (2012)	****	*	*	6
Daraniyagala et al. (2019)	****	*	**	7
Altun et al. (2010)	****	**	**	8
Nzomiwu et al. (2022)	***	**	**	7
Hadeya et al. (2017)	***	*	***	7
Vandal et al. (2018)	**	**	***	7
Jawed et al. (2020)	****	*	***	8
Aburahma et al. (2021)	***	**	**	7

### Meta-analysis

#### The pooled prevalence of dental caries among children with cerebral palsy

**Fig. 2:**
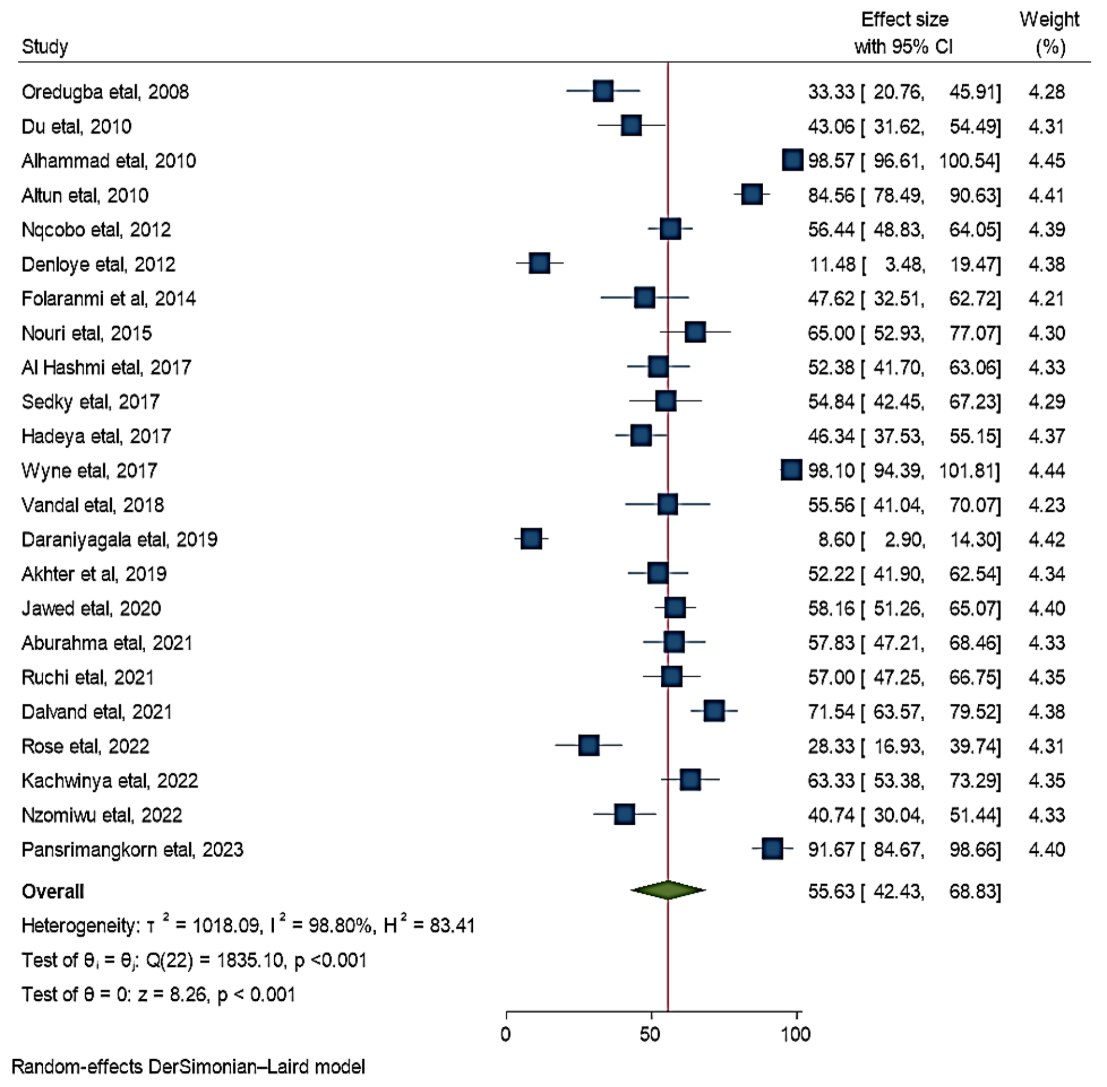
Forest plot of pooled prevalence of dental caries among children with cerebral palsy in 17 countries from Africa and Asia, 2023 (*n* = 23)

In this study, the pooled estimate is considered from 23 original articles that reported the prevalence of dental caries among children with cerebral palsy. Overall, the pooled prevalence of dental caries in Africa and Asia among children with cerebral palsy was 55.6% (95% CI: 42.4, 68.8). There was statistically significant heterogeneity among studies (I^2^ = 98.8%, *P*-value < 0.001) (Fig. 2).

### Heterogeneity management

In the random effects model, there was statistically significant heterogeneity among studies (I^2^ = 98.8%, *P*-value < 0.001). As a result, sub-group and sensitivity analysis were used to handle this heterogeneity.

#### Subgroup analysis

**Fig. 3:**
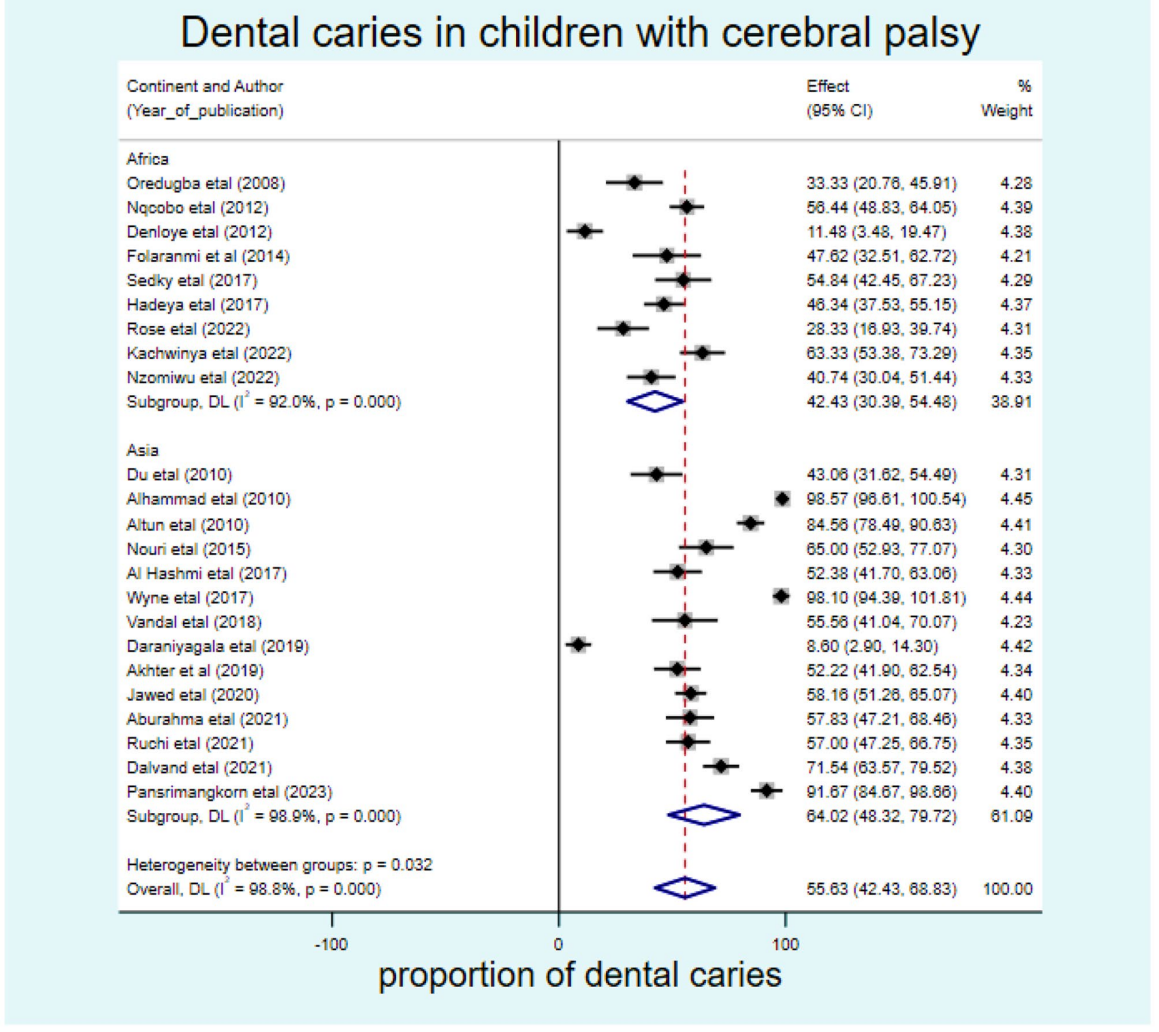
Sub-group pooled prevalence of dental caries in children with cerebral palsy in 17 countries from Africa and Asia, 2023 (*n* = 23)

Sub-group analysis was done to see the pooled estimate of dental caries among children with cerebral palsy by continent. Hence, the pooled prevalence of dental caries among African children with cerebral palsy was 42.43% (95% CI: 30.39, 54.58). The pooled prevalence was slightly higher in Asia with 64% (95% CI: 48.32, 79.72) (Fig. 3).

#### Sensitivity analysis

The sensitivity analysis revealed that there was no study that extensively affected the pooled prevalence of dental caries among children with cerebral palsy in Africa and Asia, as shown in the supplementary file (S. Fig. 1).

#### Small study effect

**Fig. 4:**
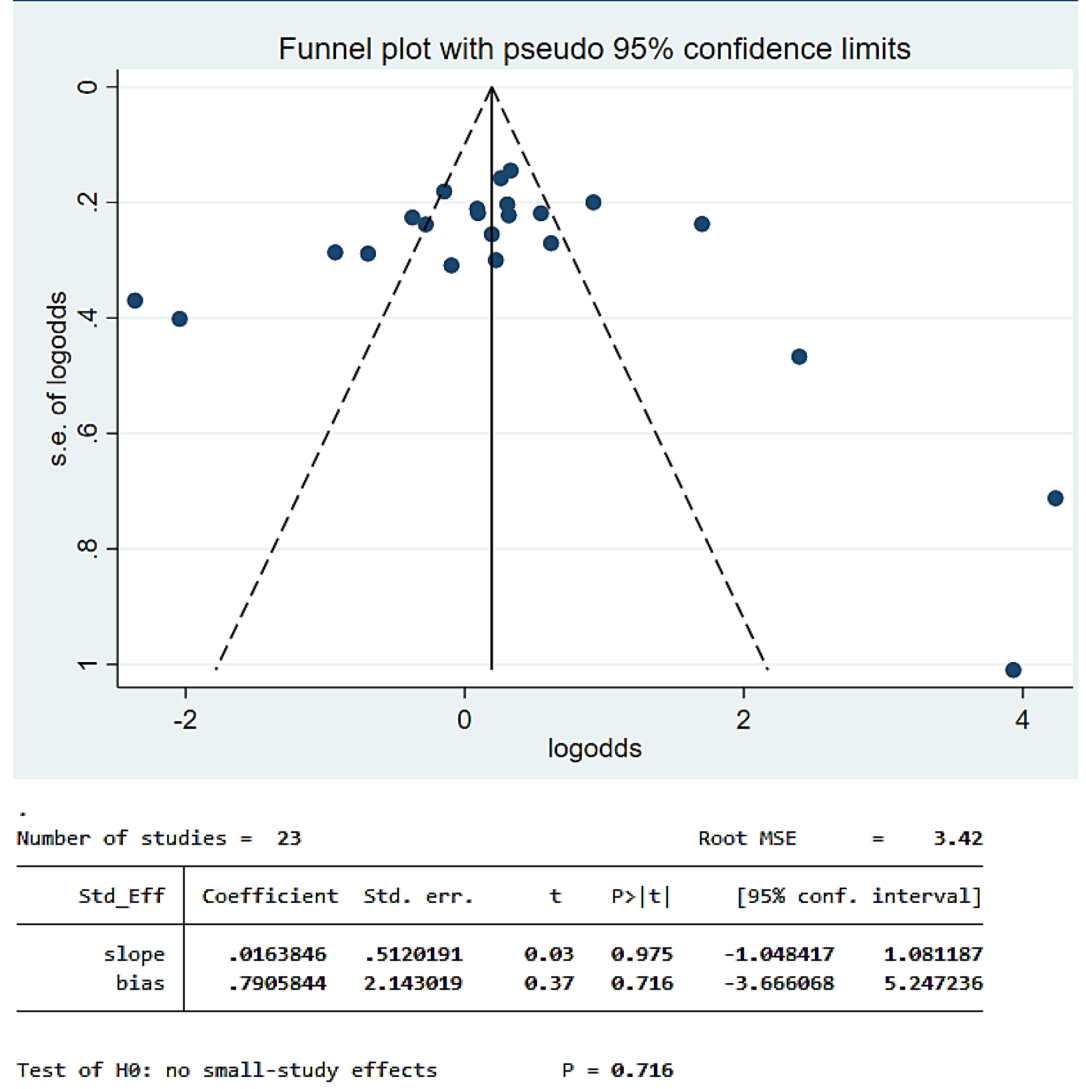
Funnel plot for dental caries in children with cerebral palsy in 17 countries from Africa and Asia, 2023 (*n* = 23)

The existence of a small study effect was checked by using funnel plots and Egger’s test from the log odds scale of the proportion. Hence, the funnel plot showed a symmetric distribution, and Egger’s test was 0.716, indicating there was no publication bias (Fig. 4).

### The mean DMFT value of children with cerebral palsy

**Fig. 5:**
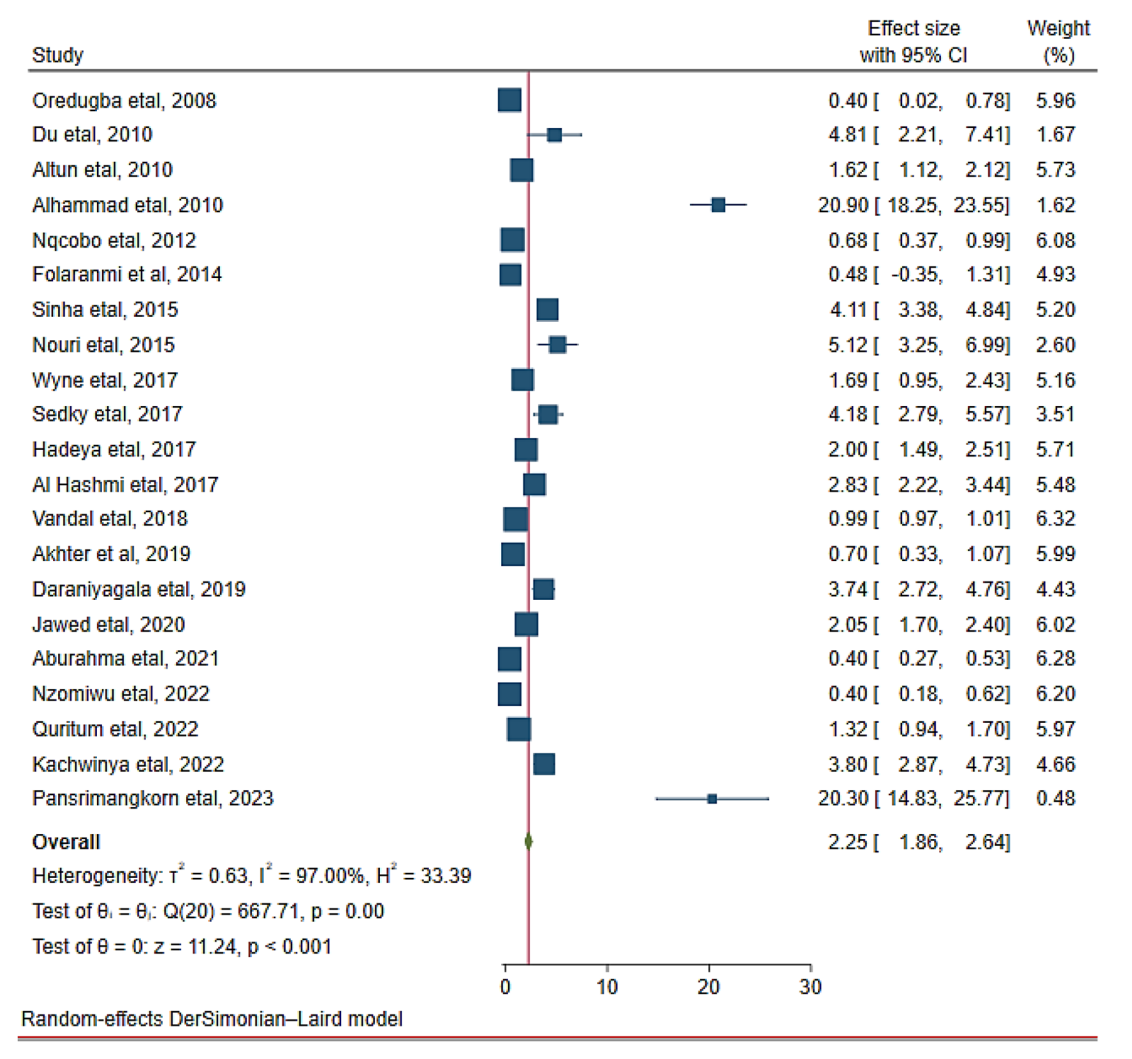
Pooled mean DMFT of children with cerebral palsy in 17 countries from Africa and Asia, 2023 (*n* = 21)

**Fig. 6:**
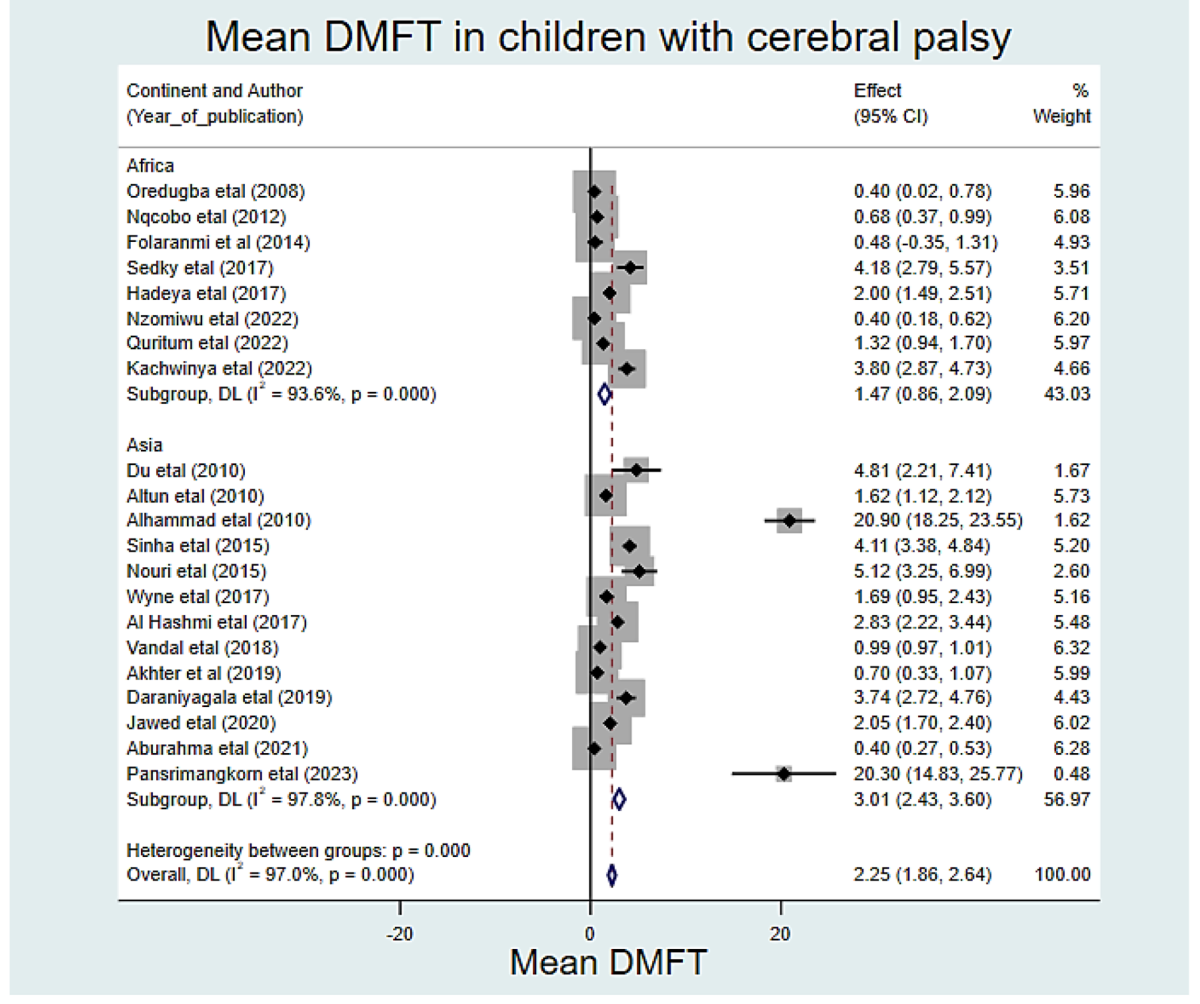
Sub-group pooled mean DMFT of children with cerebral palsy in 17 countries from Africa and Asia, 2023 (*n* = 21)

**Fig. 7:**
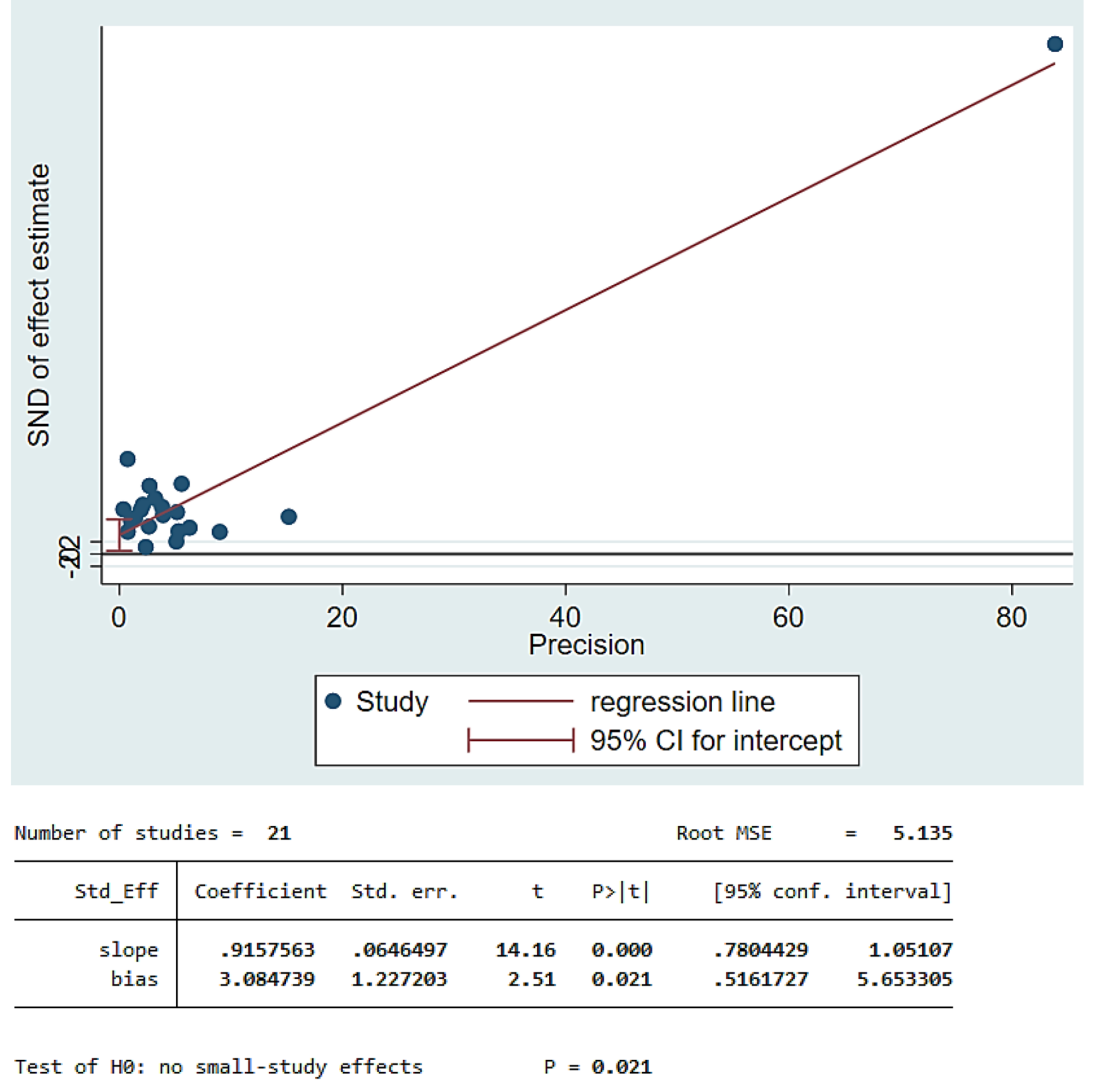
Funnel plot for mean DMFT of children with cerebral palsy in 17 countries from Africa and Asia, 2023 (*n* = 21)

**Fig. 8:**
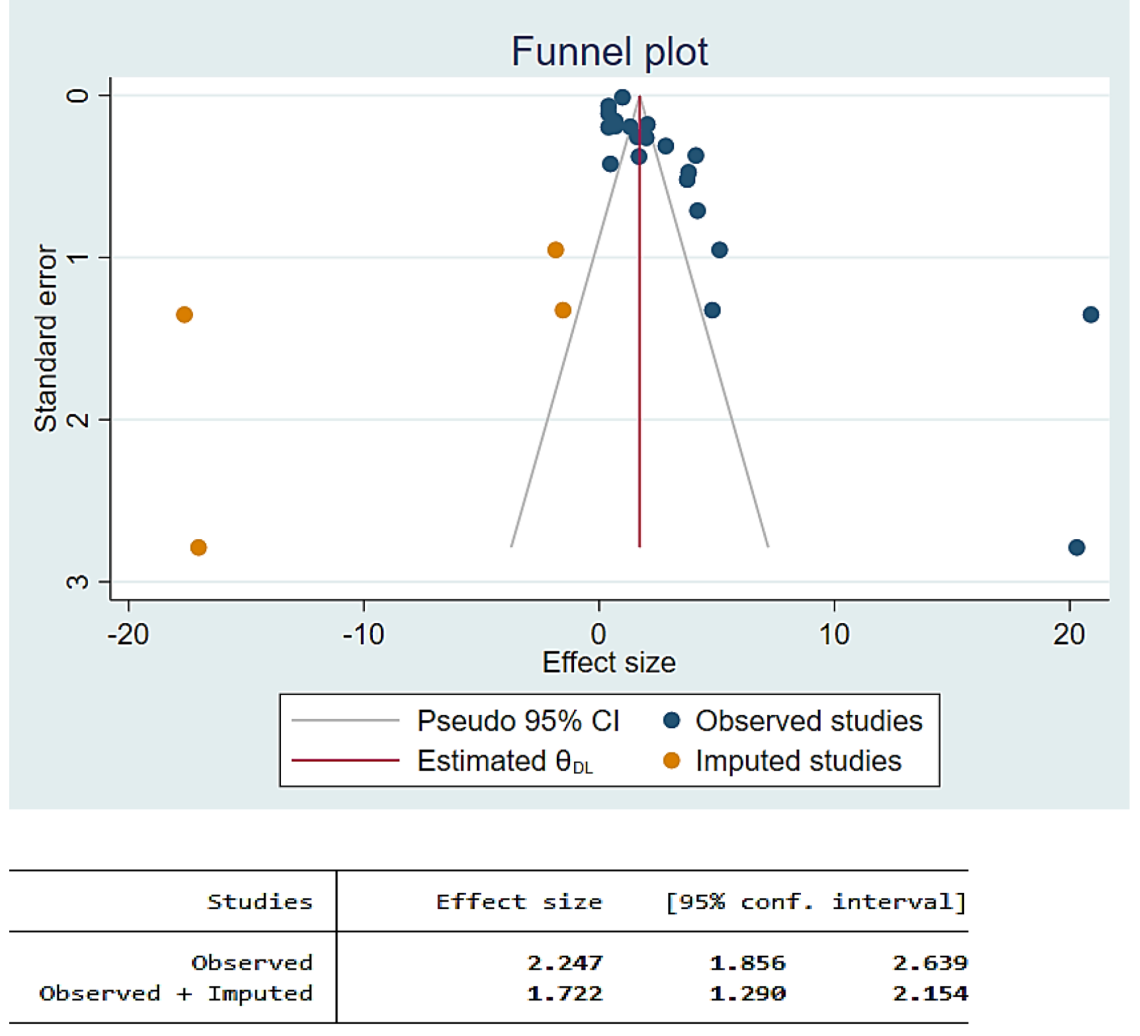
Fill and trim analysis for mean DMFT of children with cerebral palsy in 17 countries from Africa and Asia, 2023 (*n* = 21)

In the random effects model analysis, the pooled mean DMFT of dental caries in children with cerebral palsy was 2.25 (95% CI: 1.86, 2.64). The highest weight among studies was observed from the studies conducted by Vandal et al. [37], Aburahma et al. [45], Nzomiwu et al. [24]. In the pooled estimate of mean DMFT, there was statistically significant heterogeneity among studies (I^2^ = 97.0%, *p*-value < 0.001) (Fig. 5). Hence, a sub-group analysis showed that the pooled mean DMFT in Africa was 1.47 (95% CI: 0.86, 2.09). Similarly, the mean DMFT in Asia was 3.01 (95% CI: 2.43, 3.60) (Fig. 6). Egger’s test revealed that there was a small study effect (*p*-value = 0.021) (Fig. 7). As a result, a fill and trim analysis was considered to estimate the potentially missing studies due to publication bias in the funnel plot and to adjust the overall effect of the estimate (Fig. 8).

## Discussion

Prioritizing oral hygiene for children with cerebral palsy is essential as these groups of children have unique oral health needs and their overall health and quality of life be improved with optimal oral hygiene and dental care [46]. Hence, it is useful to design more extensive preventative measures and dental therapy for children who are at high risk of acquiring dental caries. As a result, this systematic review and meta-analysis aimed to determine the pooled prevalence of dental caries and pooled mean DMFT among children with cerebral palsy based on 25 studies from 17 countries in Africa and Asia.

In this review, the pooled prevalence of dental caries among children with cerebral palsy was 55.6% across Africa and Asia. This is similar to a study conducted in Brazil [14, 47]. However, the finding from the current study was higher than the findings reported from Mexico [48] and Teresina, Piaui, Brazil [49]. this variation might be due to the current study is a pooled study from two continents. The findings of this review and other studies conducted in Brazil and Mexico indicate that poor oral hygiene habits are observed in children with CP all over the world, suggesting that this is a worldwide problem. This high burden of dental caries in these groups of children might be due to their physical disabilities may limit their ability to practice proper oral hygiene, such as brushing and flossing, leading to plaque buildup and tooth decay [50]. Additionally, some children with cerebral palsy may have difficulty chewing and swallowing, resulting in higher consumption of soft or sweetened foods that contribute to tooth decay. Therefore, it is vital to provide specific dental treatment and support to address these particular challenges and limit the impact of dental caries in children with cerebral palsy [51].

From the sub-group analysis, the present study revealed that the pooled prevalence of dental caries among children with cerebral palsy in Africa was 42.43% and a slightly higher magnitude of 64% in Asia. The possible explanation could be; that the number of studies considered was different, as only nine studies in Africa were included. Additionally, countries with lower socioeconomic status often have a higher burden of dental caries. This is because individuals may have limited access to preventive dental care, have poor oral hygiene practices, and consume diets high in sugar [31]. Moreover, the availability and effectiveness of oral health policies and practices vary across countries. Some countries may have better preventive measures, such as community water fluoridation, dental sealant programs, and school-based oral health education, which can help reduce the burden of dental caries.

In this study, the pooled mean DMFT of dental caries in children with cerebral palsy was 2.25. This is higher than findings from previous studies [47, 49, 52, 53]. On the other hand, it was found to be lower than a study conducted in Brazil [46] and Croatia [54]. This variation could be partially due to the current study being based on pooled data obtained from seventeen countries in Africa and Asia. This study also revealed that the pooled mean DMFT was 1.47 and 3.01 in Africa and Asia respectively. These differences across the regions and countries might be due to variations in better access to dental care, healthcare infrastructure, and affordability of treatment. Besides, variation in dental caries might be due to differences in; sugar levels of diet, countries with diets high in sugar, frequency of snacking, and consumption of preventive foods such as fruits and vegetables across countries.

Overall, a combination of socioeconomic, cultural, policy, and behavioral factors affect the prevalence of dental caries across countries. The burden of dental caries can be decreased globally by addressing these issues through better oral health policies, access to care, education, and awareness targeting these groups of disadvantaged people.

### Limitations of the study

This review is a descriptive study focused on providing the magnitude of dental caries, which may lack in-depth analysis and fail to provide detailed insights into the relationship between risk factors with dental caries. Besides, articles published only in the English language were included. Moreover, findings in this study were obtained only from 25 studies in 17 countries. Therefore, it is important to consider these limitations when interpreting the findings. Lastly, we recommend analytical studies to identify factors affecting dental health conditions in children with cerebral palsy using data from a broader setting. Furthermore, conducting longitudinal studies to assess the long-term impact of oral health interventions, as well as exploring the experiences of both children with cerebral palsy and their caregivers can provide valuable insights.

## Conclusion

In this study, dental caries was found to be unacceptably high in children with cerebral palsy. Nearly half of the children with cerebral palsy in these settings had dental caries. Almost six in ten children with cerebral palsy in Asia had dental caries. Hence, oral health promotion initiatives should target children with CP, and this group of children must receive early preventive dental care.

### Electronic supplementary material

Below is the link to the electronic supplementary material.

Supplementary Material 1

## Data Availability

The extracted data is available from the corresponding author and can be given on request.
