# Supplementary material for: Dental caries and mean values of DMFT among children with cerebral palsy: a systematic review and meta-analysis
Source: BMC Oral Health. 2024 Feb 15;24:241. doi: 10.1186/s12903-024-03985-5 (PMC10868010; doi:10.1186/s12903-024-03985-5)
Supplement: Supplementary file 1 — Supplementary Material 1 [file 12903_2024_3985_MOESM1_ESM.docx]

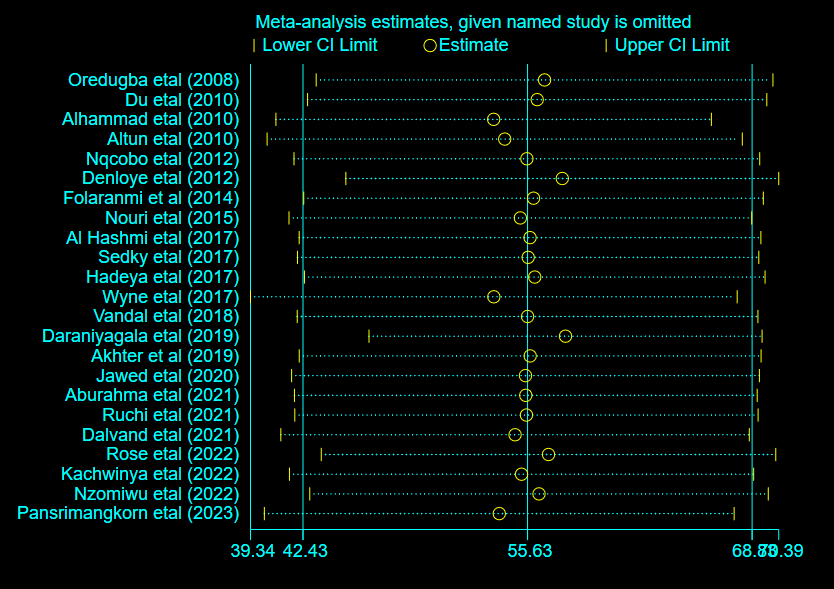


S. Figure 1: Sensitivity analysis plot for the pooled prevalence of dental caries among children with cerebral palsy in Africa and Asia, 2023 (n=23).
